# Therapeutic Effects of Autonomic Sensory Meridian Response in Orthodontic Practice: Enhancing Patient Comfort and Well-Being

**DOI:** 10.7759/cureus.74914

**Published:** 2024-12-01

**Authors:** Ipsita Roy, Sanghamitra Jena, Nivedita Sahoo, Debanwita Dutta, Soumayan Mondal

**Affiliations:** 1 Orthodontics and Dentofacial Orthopaedics, Kalinga Institute of Dental Sciences, Bhubaneswar, IND; 2 Prosthodontics and Crown &amp; Bridge, Kalinga Institute of Dental Sciences, Bhubaneswar, IND; 3 General Medicine, Kalinga Institute of Medical Sciences, Bhubaneswar, IND

**Keywords:** asmr, auditory stimuli, dental clinic management, innovative approach, oddly satisfying internet content, orthodontic, patient comfort, patient relaxation, practice management, visual stimuli

## Abstract

Autonomous sensory meridian response (ASMR) is a sensory phenomenon characterized by tingling sensations triggered by specific auditory or visual stimuli, offering a novel approach to anxiety reduction and relaxation. This review explores the therapeutic potential of ASMR in orthodontics by examining its physiological and psychological benefits, neuroscientific basis, and its potential to enhance patient comfort, manage dental anxiety, and improve communication within orthodontic practice. A comprehensive literature review was conducted to evaluate studies on ASMR's physiological, psychological, and clinical effects, focusing on ASMR-induced relaxation, patient-specific triggers, and its integration into orthodontic settings. ASMR has been shown to lower heart rates, improve mood, and reduce stress across clinical and non-clinical populations. Functional MRI (fMRI) studies reveal brain activation in regions associated with reward and emotional processing during ASMR experiences.

ASMR’s impact on dental anxiety is promising, with studies suggesting its potential to distract patients and foster a sense of calm during procedures. Patient-specific triggers, such as whispering and tapping, can be tailored to individual preferences, offering a non-invasive, cost-effective method for anxiety reduction in orthodontic patients. Incorporating ASMR content during procedures and adopting ASMR-inspired communication techniques may further enhance patient comfort and compliance. By inducing a meditative state, ASMR distinguishes itself from traditional distraction methods like music or television. ASMR holds significant promise as a therapeutic tool in orthodontics, potentially improving patient experiences by reducing anxiety and discomfort. Further research is warranted to investigate its long-term effects, optimize trigger selection for specific populations, and explore its combination with other relaxation techniques.

## Introduction and background

In an era where patient-centered care is paramount, innovative approaches to enhance patient comfort and alleviate anxiety, particularly within stress-inducing clinical settings, are continually being explored. Autonomous sensory meridian response (ASMR), a distinctive sensory phenomenon known for eliciting a calming and pleasurable experience, presents a promising avenue in this pursuit. Often described as a tingling sensation or "brain massage," ASMR is typically triggered by specific auditory or visual stimuli [1]. Initially, it may seem improbable that a concept with non-scientific origins could become a workable model within cognitive sciences. However, ideas such as ASMR often first emerge in sociolinguistic contexts and later attract systematic investigation from psychologists and neuroscientists, albeit with varying degrees of success [2].

ASMR culture originated through discussion threads on health forums, eventually leading to the exchange of videos that gave users tingles on platforms like YouTube, Reddit, and Soothetube. Over time, the community evolved from sharing "unintentional" videos - content created for other purposes but effective as ASMR triggers - to producing "intentional" videos crafted by self-described "ASMRtists" [3]. Despite its origins outside conventional science, ASMR has garnered significant public interest, with millions seeking its content for relaxation, stress reduction, and sleep improvement.

This comprehensive review explores the therapeutic potential of ASMR, examines its neuroscientific underpinnings, and discusses its potential applications in orthodontics, where patient anxiety and discomfort are common concerns.

## Review

Therapeutic potential of ASMR

Physiological and Psychological Benefits

Early investigations into ASMR primarily focused on its perceived calming effects, with many enthusiasts utilizing ASMR videos for relaxation and sleep [1]. Subsequent research has provided empirical support for these anecdotal reports. For instance, Poerio et al. demonstrated that individuals experiencing ASMR showed signs of a physiological relaxation response, such as lower heart rates and higher skin conductance levels [4]. Additionally, these individuals reported significant reductions in stress and mood improvements, especially those who frequently engaged with ASMR content.

Personality Traits and ASMR

Beyond its immediate effects, research has also explored personality traits associated with ASMR susceptibility. Fredborg et al. observed that individuals prone to ASMR tend to exhibit higher scores in "openness to experience," a personality trait linked to curiosity and an inclination toward novel experiences [5]. Such individuals often use ASMR as a coping mechanism for anxiety and to enhance emotional well-being.

Distinctive Features of ASMR

While ASMR shares similarities with other atypical conscious states, it also exhibits unique characteristics. One distinction is the duration of the tingling sensations triggered by ASMR, which can last several minutes or longer, contrasting with the relatively transient nature of other sensory-emotional experiences, such as frisson [6,7].

Clinical Studies on ASMR's Impact

Several clinical studies have further investigated the physiological and psychological benefits of ASMR. Idayati et al. conducted a double-blind pre-experimental study revealing a significant reduction in heart rate and blood pressure among patients exposed to ASMR videos [8]. Smith et al. highlighted that ASMR viewers consistently report feelings of calm and comfort, often utilizing ASMR content during periods of stress or insomnia [9].

Beyond the Tingle: The Social Comfort of ASMR

Trenholm-Jensen et al. expanded the understanding of ASMR beyond its physiological effects, suggesting that it also fosters a socially comforting environment [10]. Their qualitative study identified three primary themes: the diversity of ASMR triggers, the perception of unique intimacy facilitated by ASMR, and its role in emotional release and self-soothing.

Triggers Behind ASMR Experiences

**Figure 1:**
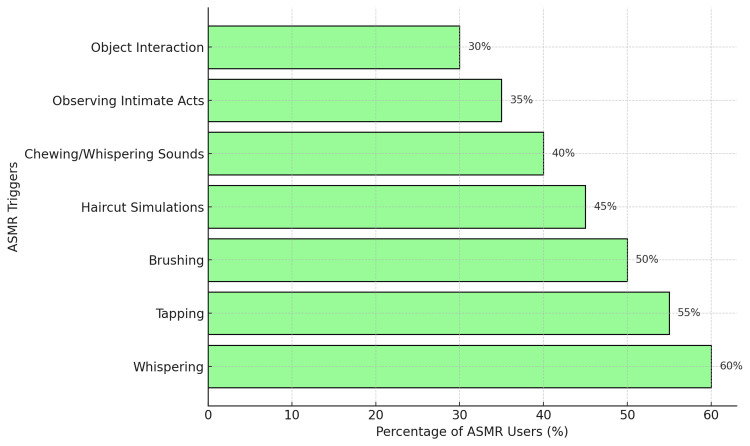
Types of ASMR Triggers and Their Popularity This bar chart displays the relative popularity of various ASMR triggers, including whispering, tapping, and personal attention, based on user studies. Whispering and tapping are particularly favored among ASMR enthusiasts, highlighting their calming effects, which could be beneficial in orthodontic settings. ASMR, autonomous sensory meridian response. Refs. [3,8].

Specific stimuli reliably trigger the ASMR experience, distinguishing it from related phenomena. Research has identified prevalent triggers, including whispering, haircut simulations, tapping, scratching, and observing someone touching another's hair [7]. These triggers can be broadly categorized into five primary groups: observing individuals interacting with objects, witnessing socially mundane yet intimate acts, hearing calm repetitive sounds, experiencing simulated social encounters, and hearing chewing or whispering sounds [11]. A recent study on trigger preferences indicated that whispering and "watching people do things in a careful way" were the most favored, followed by "close personal attention" [12]. Figure 1 illustrates different types of ASMR triggers.

The Role of Empathy

Janik et al. identified a strong correlation between ASMR and "Empathetic Concern," suggesting that a heightened ability to process socially relevant information may be a prerequisite for experiencing ASMR [13]. Table 1 presents findings from a literature search for greater clarity.

**Table 1:** Summary of Key Research Findings on ASMR This table summarizes key studies on ASMR, detailing sample sizes, methodologies, outcomes, and main conclusions. ASMR, autonomous sensory meridian response; fMRI, functional magnetic resonance imaging. Data is adapted from studies by Barratt & Davis [1], Poerio et al. [4], Fredborg et al. [5], Idayati et al. [8], Smith et al. [9], Trenholm-Jensen et al. [10], Fredborg et al. [7], Roberts et al. [12], and Lochte et al. [14].

Study	Design	Sample Size	Key Outcomes	Main Conclusions
Barratt & Davis (2015) [1]	Qualitative	Not specified	Subjective reports	ASMR as a flow-like mental state
Poerio et al. (2018) [4]	Experimental	112	Decreased heart rate, increased skin conductance, reduced stress, improved mood	ASMR induces physiological relaxation and positive affect
Fredborg et al. (2018) [5]	Correlational	291	Association between ASMR and "openness to experience" personality trait	Individuals prone to ASMR tend to be more open to new experiences
Idayati et al. (2021) [8]	Pre-experimental	30	Decreased heart rate, blood pressure	ASMR videos can reduce physiological arousal
Smith et al. (2019) [9]	fMRI	10	Brain activation in reward, emotional processing, and social bonding areas	ASMR triggers pleasure and contentment
Trenholm-Jensen et al. (2022) [10]	Qualitative	20	Themes of diverse triggers, intimacy, and emotional release	ASMR provides social comfort and self-soothing
Fredborg et al. (2017) [7]	Survey	284	Identification of five main trigger categories	ASMR triggers cluster into distinct groups
Roberts et al. (2021) [12]	Correlational	133	Association between ASMR and "Empathetic Concern"	Empathy may be linked to ASMR susceptibility
Lochte et al. (2018) [14]	fMRI	10	Brain activation in reward and emotional processing areas during tingling	Tingling sensation linked to specific brain responses

Brain Imaging Studies

**Figure 2:**
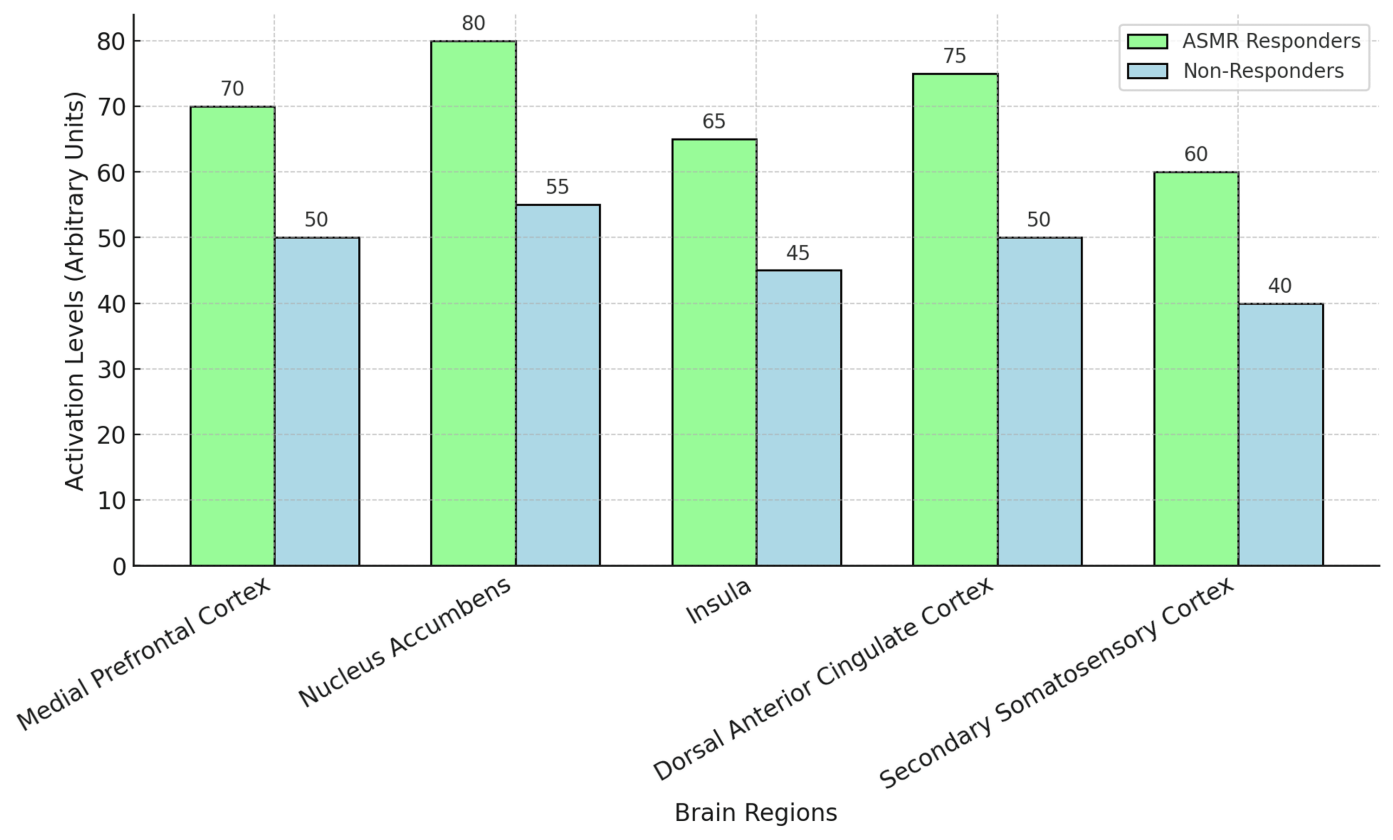
Brain Activity Comparison - ASMR Responders vs. Non-Responders This bar chart illustrates the comparison of brain activity levels across various regions, including the mPFC and NAc, between individuals who respond to ASMR stimuli and those who do not. Higher activation in ASMR responders suggests enhanced engagement with ASMR triggers, supporting their potential anxiety-reducing effects in clinical settings. ASMR, autonomous sensory meridian response; mPFC, medial prefrontal cortex; NAc, nucleus accumbens. Refs. [4,5].

While the subjective experiences associated with ASMR are well-documented, the underlying neural mechanisms remain an area of active inquiry. Initial studies employing functional MRI (fMRI) have highlighted the brain regions implicated in ASMR. Task-positive fMRI studies reveal that ASMR videos activate brain areas such as the medial prefrontal cortex and nucleus accumbens, linked to reward, emotional processing, and social bonding [14,15]. Additionally, brain activations in the insula, dorsal anterior cingulate cortex, nucleus accumbens, secondary somatosensory cortex, and supplementary motor area become more prominent when individuals who report ASMR experience the characteristic tingling sensation (Figure 2) [15].

The Default Mode Network and Neurochemical Release

ASMR also appears to engage the default mode network (DMN), a network of brain areas associated with introspection, mind-wandering, and self-referential thought [7]. The DMN is typically active during rest and relaxation, and its engagement during ASMR may help explain the feelings of calmness and mental tranquility commonly reported. Furthermore, the tingling sensation linked to ASMR may correlate with the release of neurotransmitters like oxytocin or endorphins, which are associated with pain relief, pleasure, and social bonding [4].

ASMR in orthodontic patients: Addressing specific challenges

Orthodontics presents unique challenges in ensuring patient comfort and managing anxiety. Procedures such as separator placement, bonding, wire adjustments, and routine check-ups can induce anxiety due to sounds, sensations, and the fear associated with dental procedures. ASMR offers a potential solution by creating a more soothing and supportive environment.

Managing Dental Anxiety and Phobia

ASMR can help patients manage specific dental anxieties, such as needle phobia, gag reflex, and fear of the handpiece. By providing carefully selected ASMR content featuring calming triggers, orthodontists can facilitate patient relaxation and coping strategies. For example, gentle tapping or brushing sounds can distract patients from injection sensations, while soft whispers or nature sounds can mitigate apprehension associated with the dental drill.

Enhancing Patient Comfort During Procedures

Integrating ASMR into various orthodontic procedures can foster relaxation and reduce discomfort. During lengthy or anxiety-provoking treatments, patients may choose to listen to ASMR audio or watch videos with calming triggers. Additionally, ASMR may be used pre-procedure to alleviate anticipatory anxiety and post-procedure to address any lingering discomfort.

Improving Communication and Compliance

Using ASMR-inspired communication techniques, such as adopting a soft and gentle tone, can build trust and rapport, especially with anxious patients. This approach may lead to improved communication, heightened patient satisfaction, and better adherence to treatment plans, which are essential for successful orthodontic outcomes.

Tailoring ASMR for Different Patient Demographics

Orthodontists can tailor ASMR experiences to meet the specific needs and preferences of their diverse patient populations. For children and adolescents, engaging ASMR content can reduce anxiety and foster a positive association with treatment. For adults, calming triggers can alleviate stress, while for patients with special needs, ASMR can create a more predictable and soothing environment, potentially enhancing cooperation and reducing anxiety.

Distinctiveness of ASMR Compared to Other Distraction Methods

While traditional distraction techniques, such as music or television, are frequently used in clinical settings, ASMR offers unique advantages. Unlike passive distractions, ASMR elicits specific physiological and emotional responses. The gentle sounds, soft voices, and personalized attention often characteristic of ASMR can create a sense of intimacy and comfort, promoting feelings of safety and security. Additionally, the activation of the DMN during ASMR encourages a meditative state, enabling patients to disengage from anxiety-provoking thoughts. ASMR’s ability to induce physiological relaxation responses further distinguishes it, suggesting a direct influence on the body's stress-response mechanisms. Table 2 compares ASMR with other distraction techniques.

**Table 2:** Comparison of ASMR With Other Distraction Techniques in Dental Settings This table provides a comparative analysis of ASMR and other common distraction techniques, such as music and VR, used to alleviate dental anxiety. ASMR offers a unique multi-sensory appeal, potentially improving patient relaxation and engagement. ASMR, autonomous sensory meridian response; VR, virtual reality. Refs. [8,9].

Technique	Sensory Modalities Engaged	Level of Patient Interaction	Evidence of Effectiveness	Potential Advantages	Potential Disadvantages
ASMR	Auditory, visual, tactile (tingling)	Moderate (can be personalized)	Emerging evidence for anxiety reduction and relaxation	Multi-sensory, personalized, fosters a sense of intimacy and control	Not universally effective, may require specific triggers or preferences
Music	Auditory	Low (passive listening)	Established evidence for anxiety reduction and relaxation	Readily available, familiar to most patients	May not be engaging for all, limited personalization
Television	Auditory, visual	Low (passive viewing)	Limited evidence for anxiety reduction, primarily distraction	Readily available, familiar to most patients	May not be suitable for all procedures, limited personalization
Virtual reality (VR)	Visual, auditory, sometimes tactile	High (immersive experience)	Emerging evidence for anxiety reduction and pain management	Highly engaging, potential for distraction and relaxation	Costly, may not be suitable for all patients or procedures

Practical implementation of ASMR in orthodontics

**Figure 3:**
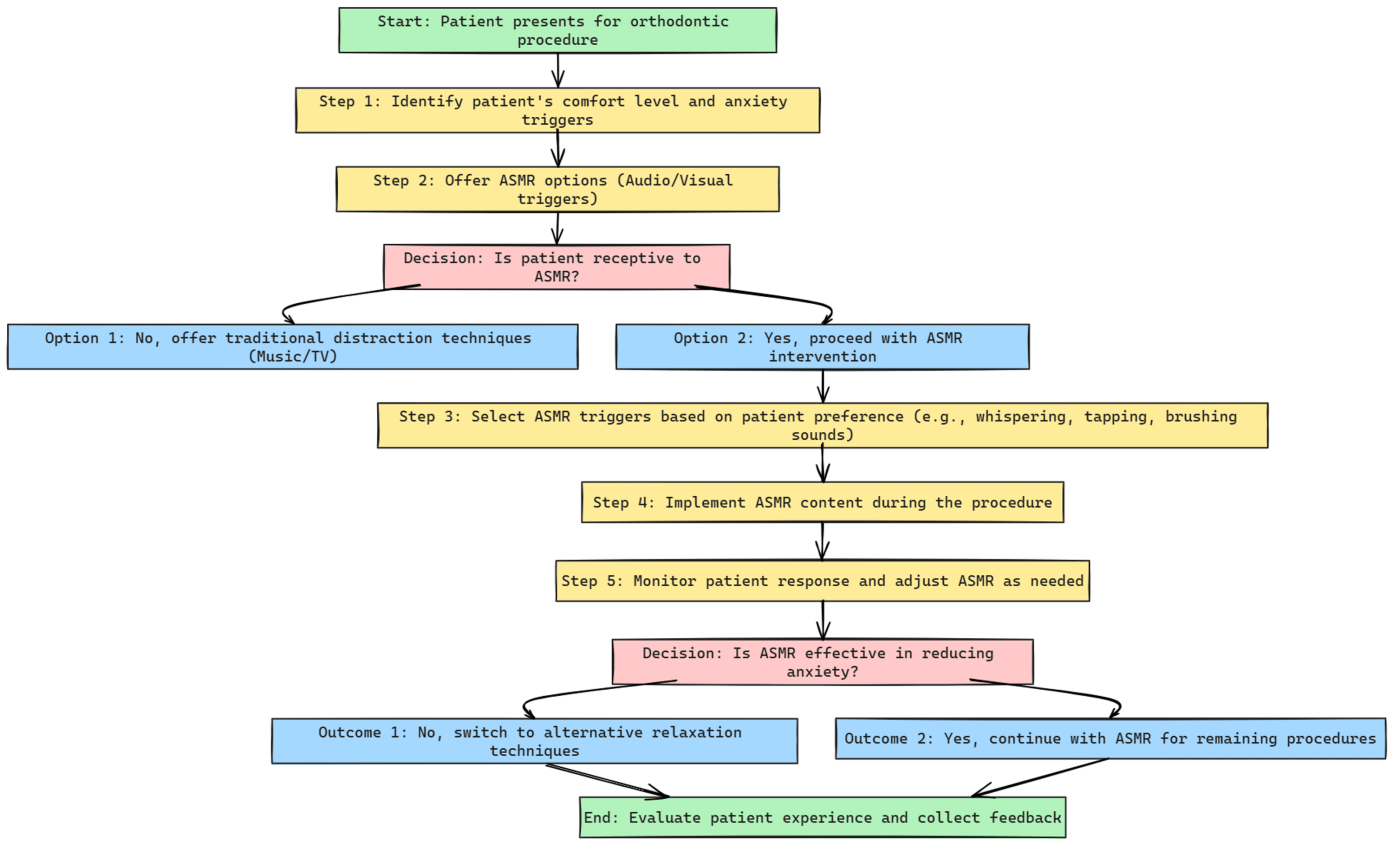
Implementation of ASMR in Orthodontic Practice: A Step-by-Step Approach This flowchart outlines a structured approach for incorporating ASMR into orthodontic practice, beginning with patient assessment, moving through trigger selection, and including feedback for adapting the ASMR experience to each patient. This tailored process ensures anxiety reduction and patient comfort are optimized. ASMR, autonomous sensory meridian response. Ref. [5].

Orthodontists can integrate ASMR into their practice through various approaches, as outlined in Table 3. This includes offering patients the option of listening to ASMR audio or watching videos during procedures, supported by noise-canceling headphones to optimize the experience. Customized ASMR playlists tailored to specific procedures based on duration and potential discomfort level can also be created. Brief ASMR sessions in the waiting room or treatment chair before procedures may help alleviate anticipatory anxiety, while ASMR-inspired communication techniques, such as maintaining a calm, soft voice and using deliberate movements, can further enhance the patient's comfort. Providing access to ASMR content following procedures may help address any residual anxiety or discomfort. Figure 3 illustrates a step-by-step approach to incorporating ASMR in orthodontic practice, emphasizing patient engagement and feedback at each stage.

**Table 3:** Practical Implementation of ASMR in Orthodontic Practice This table presents practical strategies for integrating ASMR into orthodontic treatments, including the use of noise-canceling headphones, curated ASMR playlists, and pre-treatment sessions. These strategies are designed to reduce anxiety and enhance patient comfort throughout the treatment process. ASMR, autonomous sensory meridian response; DMN, default mode network. Refs. [5,11].

ASMR Technique	Target Patient Population	Potential Benefits	Implementation Considerations
ASMR audio/video during procedures	All ages	Reduced anxiety, enhanced comfort, improved patient experience	Need for appropriate content selection, noise-canceling headphones
Customized ASMR playlists	All ages	Tailored relaxation and distraction based on procedure type and patient preferences	Requires understanding of patient needs and preferences, time investment for playlist creation
Pre-treatment ASMR sessions	Particularly beneficial for anxious patients	Reduced anticipatory anxiety, improved patient cooperation	May require additional time and space in the waiting area
ASMR-inspired communication	All ages, especially anxious patients	Enhanced trust and rapport, improved communication and compliance	Requires training and conscious effort from practitioners
Post-procedure ASMR	All ages	Management of residual anxiety or discomfort, positive association with treatment	Easy to implement, can be offered as a take-home resource

Future directions and limitations

The promising potential of ASMR to enhance patient comfort and well-being, along with key areas for future research, is illustrated in Figure 3. Although current evidence supporting ASMR’s benefits in clinical settings is encouraging, further research is essential to understand its mechanisms and optimize its application. Future studies should examine (1) the long-term efficacy of ASMR in reducing dental anxiety and improving treatment compliance; (2) the optimal types of ASMR triggers for different patient demographics and procedures; (3) the potential for combining ASMR with other relaxation techniques, such as mindfulness or guided imagery; (4) personalized ASMR experiences based on individual patient needs; and (5) the use of cutting-edge technologies like augmented reality (AR) and virtual reality (VR) to create immersive ASMR experiences.

While current evidence highlights ASMR's potential to enhance patient comfort and well-being, it is important to acknowledge existing research limitations. Many studies involve small sample sizes, and long-term research on ASMR’s efficacy in reducing dental anxiety and improving compliance is needed. Additionally, identifying optimal ASMR triggers for diverse patient populations and specific procedures requires further exploration. Future research should also explore ASMR’s synergistic potential with relaxation techniques, such as mindfulness or guided imagery. Customizing ASMR to each patient's needs, and incorporating ASMR with technologies like AR and VR, could significantly expand its therapeutic applications.

## Conclusions

ASMR represents an innovative and promising approach to enhancing patient comfort and well-being within the realm of orthodontic practice. Additionally, it is imperative to address the ethical considerations associated with utilizing ASMR in clinical settings. Obtaining informed consent from patients, ensuring the suitability of ASMR content, and respecting individual preferences are paramount to ethical implementation.

The existing evidence underscores its considerable potential as a non-invasive, cost-effective, and readily accessible intervention for enhancing patient well-being. Through the judicious integration of ASMR into their practice, orthodontists can cultivate a more empathetic and supportive environment, fostering a sense of tranquility and empowerment among their patients.
